# Reperfusion Criteria in Patients Submitted to Fibrinolysis: Is There Room for Improvement?

**DOI:** 10.5935/abc.20180245

**Published:** 2019-01

**Authors:** Milena Soriano Marcolino, Antonio Luiz Pinho Ribeiro

**Affiliations:** 1 Faculdade de Medicina e Centro de Centro de Telessaúde do Hospital das Clínicas - Universidade Federal de Minas Gerais, Belo Horizonte, MG - Brazil

**Keywords:** ST Elevation Myocardial Infarction/mortality, Percutaneous Coronary Intervention/economics, Fibrinolysis, Thrombolytic THerapy/methods, Time Factors, Electrocardiography/methods

Many ST-elevation acute myocardial infarction (STEMI) patients seek care in hospitals without percutaneous coronary intervention (PCI) capability and cannot be submitted to PCI within the guideline-recommended timelines, and, instead, they are often submitted to fibrinolysis as the initial reperfusion therapy. Rapid, simple and readily available bedside measures are of utmost importance for timely assessment of the efficacy of reperfusion therapy early after fibrinolysis in acute STEMI,^1^ in order to immediately identify the ones who require rescue PCI.^2^^,^^3^

In an editorial for Circulation in 2001, Gibson^4^ stated “In a time of dizzying advances in diagnostic modalities, it is refreshing to see what a useful, simple, noninvasive, broadly accessible, easily repeatable/applied, and affordable tool the electrocardiography (ECG) is”.^4^ This is still up to date. Multiple studies have demonstrated improved outcomes among patients who achieve complete ST resolution at 60-90 minutes after fibrinolytic therapy, and it is recommended that the absence of > 50% reduction in ST elevation in the worst lead at 60-90 minutes should prompt strong consideration of coronary angiography and rescue PCI. ^2^^,^^3^ However, this measure, combined with the absence of reperfusion arrhythmias at 2 hours after treatment, has a positive predictive value of 87% and a negative predictive value of 83% to predict failure of reperfusion, ^2^^,^^5^ indicating that there is still room for improvement in accuracy.

In the well-structured analysis by Dotta et al.^6^ in the article “*Regional QT Interval Dispersion as an Early Predictor of Reperfusion in Patients with Acute Myocardial Infarction after Fibrinolytic Therapy*”, published in this Arquivos Brasileiros de Cardiologia issue,^6^ the results reinforced Gibson’s statement. The authors assessed the performance of QT interval dispersion in addition to classical reperfusion criteria as an early marker of reperfusion in 104 STEMI patients from emergency care units in Sao Paulo who underwent fibrinolysis with tenecteplase (TNK).

The concept of QT interval dispersion was introduced in the 1990s, as a non-invasive method for the detection of ventricular repolarization heterogeneity, and previous studies have shown that reduction of QT interval dispersion post-thrombolysis was an independent predictor of coronary reperfusion.^7^ Dotta et al.^6^ study was the first one to assess QT interval dispersion in STEMI patients who underwent pharmaco-invasive strategy. Interestingly, the authors observed an increase in regional dispersion of corrected QT interval 60 minutes after TNK in anterior wall infarction in patients with angiographic findings of complete recanalization (TIMI flow 3 and Blush grade 3). When they added regional QTcD to electrocardiographic criteria for reperfusion, the area under the receiving operating characteristic curve (ROC) changed from 0.81 (0.72-0.89) to 0.87 (0.78-0.96), demonstrating an improved discriminatory ability.^6^

Some limitations should be pointed out and most of them are recognized by the authors. This measure was not tested in patients with bundle branch block, atrial fibrillation or previous myocardial infarction, as those could compromise the QT interval dispersion assessment. Although a good concordant agreement was noted between examiners (kappa coefficient = 0.84),^6^ errors in manual measurement of QT intervals are common^8^ and, in the real world, there are consistent differences in the measurements between cardiologists, what can compromise the acuity of the evaluation of the QT dispersion, especially in an emergency situation as the management of the myocardial infarction.

To overcome these limitations, the authors commented about the need to advance in the methodology to measure QT interval and ventricular repolarization. The use of computerized programs for automated ECG interpretation has shown good accuracy levels for ECG interval measurements,^9^^,^^10^ and it might improve regional QT dispersion assessment. More than ever, development of computerized automatic calculation and studies in different populations, with a larger sample size, are needed to allow the external validation of including regional QT dispersion together with traditional reperfusion criteria in reperfusion assessment after fibrinolysis.

## References

[1] de Lemos JA, Braunwald E (2001). ST segment resolution as a tool for assessing the efficacy of
reperfusion therapy. J Am Coll Cardiol.

[2] O'Gara PT, Kushner FG, Ascheim DD, Casey Jr DE, Chung MK, de Lemos JA (2013). 2013 ACCF/AHA guideline for the management of ST-elevation
myocardial infarction: a report of the American College of Cardiology
Foundation/American Heart Association Task Force on Practice
Guidelines. J Am Coll Cardiol.

[3] Ibanez B, James S, Agewall S, Antunes MJ, Bucciarelli-Ducci C, Bueno H (2018). 2017 ESC Guidelines for the management of acute myocardial
infarction in patients presenting with ST-segment elevation: The Task Force
for the management of acute myocardial infarction in patients presenting
with ST-segment elevation of the European Society of Cardiology
(ESC). Eur Heart J.

[4] Gibson CM (2001). Time is myocardium and time is outcomes. Circulation.

[5] Sutton AG, Campbell PG, Price DJ, Grech E, Hall J, Davies A (2000). Failure of thrombolysis by streptokinase: detection with a simple
electrocardiographic method. Heart.

[6] Dotta G, Fonseca FAH, Izar MC, de Souza MT, Moreira FT, Pinheiro LFM (2019). A dispersão do intervalo QT regional como preditor precoce
de reperfusão em pacientes com infarto agudo do miocárdio
pós-terapia fibrinolítica. Arq Bras Cardiol.

[7] Lopes NH, Grupi C, Dina CH, de Gois AF, Hajjar LA, Ayub B (2006). QT interval dispersion analysis in acute myocardial infarction
patients: coronary reperfusion effect. Arq Bras Cardiol.

[8] Murray A, McLaughlin NB, Bourke JP, Doig JC, Furniss SS, Campbell RW (1994). Errors in manual measurement of QT intervals. Br Heart J.

[9] Salerno SM, Alguire PC, Waxman HS (2003). Competency in interpretation of 12-lead electrocardiograms: a
summary and appraisal of published evidence. Ann Intern Med.

[10] Willems JL, Abreu-Lima C, Arnaud P, van Bemmel JH, Brohet C, Degani R (1991). The diagnostic performance of computer programs for the
interpretation of electrocardiograms. N Engl J Med.

